# Emotional Dysregulation and Sleep Problems: A Transdiagnostic Approach in Youth

**DOI:** 10.3390/clinpract14030074

**Published:** 2024-05-21

**Authors:** Gianluca Sesso, Fulvio Guccione, Simone Pisano, Elena Valente, Antonio Narzisi, Stefano Berloffa, Pamela Fantozzi, Valentina Viglione, Annarita Milone, Gabriele Masi

**Affiliations:** 1 Social and Affective Neuroscience Group, Molecular Mind Laboratory, IMT School for Advanced Studies, 55100 Lucca, Italy; 2 Department of Child and Adolescent Psychiatry and Psychopharmacology, IRCCS Stella Maris Foundation, 56128 Pisa, Italy; 3 Department of Translational Medical Sciences, Child and Adolescent Neuropsychiatry, University of Naples Federico II, 80138 Naples, Italy

**Keywords:** sleep, emotional dysregulation, adolescents, youth

## Abstract

Background: Sleep is a complex phenomenon that affects several aspects of life, including cognitive functioning, emotional regulation, and overall well-being. Sleep disturbances, especially during adolescence, can negatively impact emotional regulation, making it a critical factor in targeting psychopathology. Methods: This study explores the interplay between emotional dysregulation (ED) and sleep patterns in a sample of 90 adolescent patients by means of self- and parent-rated clinical measures. Results: Our findings reveal a bidirectional relationship between ED and sleep problems. Adolescents with higher affective instability experience poorer sleep quality, while those with worse sleep quality exhibit higher internalizing problems. Additionally, emotional reactivity is associated with disrupted circadian rhythms. Conclusions: These results emphasize the significance of addressing sleep problems in the context of psychopathology treatment, potentially leading to improved outcomes. Further research is needed to determine the most effective treatment strategies, including nonpharmacological and pharmacological interventions. Understanding the intricate relationship between sleep problems and emotion regulation offers insights for more targeted and effective treatment approaches for youths struggling with ED.

## 1. Introduction

Sleep is a complex phenomenon that occupies approximately one-third of our lives and regulates all bodily functions, thus playing a vital role for our survival. During sleep, brain activity significantly differs from wakefulness, and such change is related to the multiple complex functions that sleep serves, often disrupted by sleep deprivation. During childhood and adolescence, a reduction in total sleep time negatively affects memory consolidation, working memory, and executive control, affecting performance in complex tasks that involves such higher brain functions [1,2]. Sleep deprivation, commonly encountered among adolescents, significantly affects overall health, mood, and academic performance, in a period of life vulnerable to changes in physical development and brain systems responsible for emotional regulation [3].

While evidence suggests a strong connection between sleep and emotional regulation, the impact of sleep loss on emotional regulation shortfalls has been less investigated [4,5]. It has been demonstrated that sleep loss leads to functional alterations in brain regions involved in emotional regulation, with an increase in subcortical reactivity and a reduction in prefrontal control over limbic structures, preventing a balanced integration of emotional signals [5]. Sleep, particularly REM sleep, contributes to consolidating conditioned fear responses, promoting better discrimination between threatening and non-threatening stimuli [6]. Due to its properties, REM sleep seems to play a key role in emotional memory consolidation, with a specific role in processing memories after emotionally significant experiences [7,8].

For this reason, a dual function of REM sleep has been proposed, which has intriguing implications for psychiatric conditions, such as major depression and post-traumatic stress disorder, in which an unbalanced REM sleep pattern is typically found [9]. First, REM sleep allows recalling experiences from wakefulness, thus reducing the emotional charge associated with intensely emotional memory traces. Second, through variations in adrenaline levels that occur during REM sleep in the brainstem and limbic and prefrontal regions, it is possible to discriminate the emotional salience of an event, thus implementing adaptive strategies congruent with the stimulus and avoiding exaggerated reactions to non-threatening situations [4,5].

A noteworthy point is that NREM sleep has also been investigated as a potential contributor to the maintenance of the functional homeostasis in humans. Recent evidence, in line with the “synaptic homeostasis hypothesis”, suggests that sleep plays an important role in cognitive and motor learning, particularly in memory consolidation and neural plasticity [10,11]. Nonetheless, slow wave-like activities during wake have detrimental effects on emotional suppression and affect regulation strategies in healthy subjects [12]. Indeed, disrupted NREM sleep has been repeatedly demonstrated in association with psychiatric disorders and neurodevelopmental conditions [13,14] and electrophysiological features of NREM sleep have also been investigated as early biological markers of psychopathology [15,16].

Sleep disorders, as emotional dysregulation (ED), can be considered transdiagnostic processes as they often occur in comorbidity with most psychiatric disorders [17], contribute to the genesis of symptoms and associated functional impairment, and share similar pathogenetic mechanisms [18]. Emotional dysregulation, expressed by symptoms of hyperarousal, excitability, mood shifts, and irritability with aggression propensity, is a shared feature of both internalizing and externalizing disorders, thus being a transdiagnostic manifestation of psychopathology [19]. Alterations in the sleep/wake cycle are a diagnostic criterion for many psychiatric conditions according to the current nosographic systems, and, even when they are not part of diagnostic criteria, sleep disruption is integral to the core psychopathology in many others. Patients with panic disorder, social anxiety, schizophrenia, and so on, frequently report sleep disturbances that further impact the underlying functioning. Hence, the goal of the present study was to investigate the link between sleep problems and ED in a clinical sample of youths, by assessing the subjective quality and features of sleep and the sleep/wake cycle as well as the ED-related symptomatology and severity, in order to verify whether the associations between these aspects are clinically meaningful. More broadly, our study could further highlight the relevance of sleep problems in psychopathology, even in a transdiagnostic perspective, which may help clinicians to define the treatment targets—the underlying psychopathology, the sleep disorder, or both—and to identify effective therapeutic approaches to treat adolescents with ED and sleep problems.

## 2. Materials and Methods

### 2.1. Participants

A sample of drug-naïve patients was consecutively recruited from October 2022 to April 2023 at the Department of Child and Adolescent Psychiatry and Psychopharmacology at the IRCCS Stella Maris Foundation hospital. This study was conducted in accordance with the Declaration of Helsinki and approved by the Regional Ethics Committee for Clinical Trials of Tuscany (Pediatric Ethics Committee at Meyer Children’ Hospital of Florence; 28 September 2022, protocol code Affect2022). All participants and their parents were informed about the assessment tools, and participation in this study was voluntary. All patients were included following a thorough screening as they met all inclusion criteria listed below. Additionally, all patients were drug-naïve before recruitment. The sample comprised 90 inpatients and outpatients aged 11 to 18 years who met the diagnostic criteria for any psychopathological condition or neurodevelopmental disorder. Diagnoses were made according to the Diagnostic and Statistical Manual of Mental Disorders—Fifth Edition (DSM-5) [20], based on medical history, clinical observations, and a semi-structured interview, the Kiddie Schedule for Affective Disorders and Schizophrenia-Present and Lifetime version (K-SADS-PL) [21], administered by trained child psychiatrists to both patients and parents.

Inclusion criteria were as follows: adolescents aged 11 to 18 years; naïve to any psychotropic drug; presence of any psychopathological condition or neurodevelopmental disorder featured by ED based on DSM-5 criteria; and intellectual functioning within the normal range, as determined by the Wechsler Intelligence Scale for Children—Fourth Edition (WISC-IV) [either Full-Scale Intelligence Quotient (FSIQ) or General Ability Index (GAI) ≥ 85]. Exclusion criteria were as follows: previous or current psychotropic medication; borderline cognitive functioning or intellectual disability; and neurological conditions or neurosensory deficits (e.g., visual and auditory).

### 2.2. Measures

The clinical assessment of patients included structured and semi-structured interviews, as well as clinical questionnaires of both self- and parent-report types. Patients were asked to complete the following questionnaires to assess sleep:

- The Pittsburgh Sleep Quality Index (PSQI) [22], a self-assessment questionnaire investigating sleep quality in the last month through 19 items distributed across 7 subscales that provide both global and partial scores, showing good convergence with objective sleep measures (e.g., actigraphy) and adequate reliability in detecting sleep problems;
- The Biological Rhythms Interview of Assessment in Neuropsychiatry (BRIAN) [23], an 18-item questionnaire assessing circadian rhythm disturbances, particularly in sleep, wakefulness, social habits, and vegetative patterns;
- The Morningness Eveningness Questionnaire (MEQ), a 19-item questionnaire assessing chronotypes as defined by sleep habits and circadian rhythms based on whether individuals are more inclined to stay awake late in the night or wake up early in the morning.

Patients were also asked to complete the following questionnaires to assess ED:

- The Youth Self Report for ages 11 to 18 (YSR—11/18) [24], a widely used self-assessment measure consisting of 113 items related to emotional and behavioral problems based on self-report;
- The RIPoSt-Y questionnaire [25,26], a 31-item self-assessment measure designed to qualitatively and quantitatively explore three main dimensions of ED in adolescence, namely affective instability, emotional reactivity, and interpersonal sensitivity.

Furthermore, all parents or caregivers of participants were invited to complete two questionnaires: the Affective Reactivity Index (ARI) [27], a 6-item questionnaire investigating the presence of irritability and impulsivity in children, along with a seventh item aimed at assessing clinical severity; and the Child Behavior Checklist for ages 6 to 18 (CBCL—6/18) [24], a 118-item questionnaire providing scores for three factors (total problems, internalizing problems, and externalizing problems), eight syndrome scales, and six scales aligned with the DSM diagnostic categories. In the current study, the Dysregulation Profile Index of the CBCL—6/18 as well as the YSR—11/18 questionnaires (CBCL—DP and YSR—DP) was calculated as the sum of the T-scores of the anxious/depressed, attention problems, and aggressive behavior subscales [28,29].

In addition, parents and adolescents were administered the Kiddie Schedule for Affective Disorders and Schizophrenia for School-Aged Children Present and Lifetime Version (K-SADS-PL) [21].

### 2.3. Clinical Characteristics of the Sample

Ninety patients were included in the sample (mean age 14.02 ± 1.89 years, age range 9–18 years, 52.22% females + 47.78% males). Diagnoses included multiple comorbidities related to different psychiatric disorders and neurodevelopmental conditions; the number and percentage of patients meeting diagnostic criteria for each specific diagnosis as well as other clinical characteristics of the sample are presented in Table 1. The vast majority of our patients were diagnosed with a bipolar spectrum disorder, with frequent comorbid anxiety disorders, either isolated or multiple, and ADHD, either combined or predominantly inattentive. Intellectual functioning was globally in the average range; as expected, metacognitive competences, including working memory and processing speed, were in the lower range.

Specificities related to sleep and ED questionnaires are presented in Table 2 and Table 3, respectively. Mean scores of the PSQI and BRIAN questionnaires obtained by our patients as well as percentage of patients meeting each of the three major chronotypes identified through the MEQ are reported in Table 2. Number and percentage of patients scoring above the clinical cut-off for each ED questionnaire’s subscale are reported in Table 3.

### 2.4. Statistical Analysis

Statistical analyses were performed using RStudio^®^ (version 1.3.1093, RStudio, PBC) and MATLAB^®^ software (version R2021b, The MathWorks, Inc., Natick, MA, USA). Analysis of variance (ANOVA) was conducted to assess significant differences between group means in variables with continuous distribution, taking into account the effects of other variables as covariates (e.g., age and gender). Tukey post hoc tests were used whenever ANOVA yielded statistically significant results.

## 3. Results

### 3.1. Effects of Emotional Dysregulation on Sleep Variables

First, we conducted ANOVAs by introducing the RIPoSt-Y—AI, ER, and IS groups based on the respective clinical cut-off scores as independent variables of the model, and the scores obtained by patients in the sleep-related questionnaires as dependent variables; gender and age range (11–14 and 15–18 years old) were introduced as covariates (see Table 4). The ANOVA revealed a statistically significant effect of the RIPoSt-Y—AI group on the total score of the PSQI sleep questionnaire (F = 3.976, *p* = 0.050). Post hoc analysis showed that individuals with higher affective instability scores have higher scores in the sleep quality scale, indicating worse sleep quality, compared to those without AI. There is also a statistically significant age effect (F = 4.785, *p* = 0.033), where the post hoc analysis shows that older patients have poorer sleep quality compared to younger ones. No statistically significant effects of RIPoSt-Y–ER and IS groups, and gender on the PSQI total score were observed.

Similarly, a statistically significant effect of the RIPoSt-Y—AI group (F = 4.251, *p* = 0.044) and the RIPoSt-Y—ER group (F = 9.196, *p* = 0.0004) on the BRIAN total score was found. Specifically, the post hoc analysis revealed that patients with higher affective instability and higher emotional reactivity scores have higher scores on the BRIAN total scale, indicating greater disruption of circadian rhythms, social habits, awakening patterns, and vegetative patterns. No statistically significant effects of RIPoSt-Y—IS group, gender, and age on the BRIAN total score were observed. Instead, no statistically significant effects of RIPoSt-Y—AI, ER, and IS groups on the MEQ total score were found, nor of gender and age range. In other words, none of the explored variables affected the chronotype.

### 3.2. Effects of Sleep Quality on Clinical Variables

We then conducted ANOVAs by introducing the PSQI groups based on the clinical cut-off score as independent variables of the model, and the scores obtained by patients in the clinical questionnaires as dependent variables; gender and age range were also introduced as covariates (see Table 5). In the ANOVAs, a statistically significant effect of the PSQI group was observed on the internalizing problems scale scores of the YSR—11/18 questionnaire. The post hoc analysis revealed that patients with worse sleep quality have higher scores in the internalizing problems’ scale of the questionnaire. No statistically significant effects of sleep quality were observed on the scales of the CBCL—6/18 questionnaire, nor on the dysregulation profile and the externalizing problem scales of the YSR—11/18. No statistically significant effects of sleep quality were observed on the ARI questionnaire.

### 3.3. Effects of Chronotype on Clinical Variables

We finally conducted ANOVAs by introducing the MEQ groups (morning, intermediate and evening chronotypes) based on the clinical cut-off scores as independent variables of the model, and the scores obtained by patients in the clinical questionnaires as dependent variables; gender and age range were also introduced as covariates (see Table 6). No statistically significant effects of chronotypes were observed on the CBCL—11/18 and YSR—11/18 questionnaires. No statistically significant effects of chronotype were observed on the ARI questionnaires.

## 4. Discussion

The present study investigates the relationship between emotional dysregulation (ED) and sleep patterns in a sample of 90 adolescent patients diagnosed with multiple comorbid psychiatric disorders and neurodevelopmental conditions. This study aims to examine the complex interplay between sleep quality and habits and emotional regulation shortfalls in this clinical population, shedding light on the potential implications for the understanding and treatment of ED in adolescents.

We first examined the impact of ED dimensions on sleep variables, revealing that those patients with higher affective instability experience worse sleep quality. This finding highlights a relationship between ED and sleep quality that is based on a correlational approach. Thus, a causal link could not be established between the variables according to our results; however, it is likely that each affects the other in a mutual relationship. Similarly, patients with higher affective instability and emotional reactivity exhibited significantly higher scores in the BRIAN questionnaire, designed to assess the quality of circadian rhythms, social habits, awakening patterns, and vegetative patterns. Sleep quality is further shown to affect clinical variables, with worse sleep quality associated with higher internalizing problems.

Nonetheless, the potential mechanisms underlying the observed associations between ED and sleep are still under investigation. Different physiological or neurobiological processes have been hypothesized to mediate the impact of ED on sleep quality and vice versa. For instance, slow wave patterns have been claimed to affect emotional regulation strategies in healthy subjects. Indeed, local sleep-like activity represents the cause of failures in emotional suppression, thus offering a functional explanation for the link among sleep loss, frontal activity changes, and ED [12]. On the other hand, REM sleep, with its brain activity patterns, seems to contribute to the consolidation of conditioned fear responses [6] and emotional memory after significant experiences [7,8]. More evidence from the neuroscientific and translational perspective are insightful to shed light on this important field of research with strong clinical implications.

Our article underscores the intricate relationship between sleep quality and patterns, emotional dysregulation, and psychopathology in adolescence. As stated above, sleep plays a critical role in human life, including cognitive and motor learning, memory consolidation, and neural plasticity [1]. Its importance during development, a period marked by significant physical and brain changes, appears even more obvious in light of the present findings. Daily life habits featured by sleep deprivation, which are commonly found during adolescence, are known to adversely affect working memory, executive control, mood stability, and emotional regulation [3]. Sleep loss, indeed, leads to functional changes in brain regions associated with emotional regulation, thereby disrupting the balance between subcortical reactivity and prefrontal control over limbic structures [5]. These alterations may hinder the functional integration of emotional signals, potentially leading to emotional dysregulation.

Hence, in light of these findings, it is important to address the issue of identifying which of the following methods should be prioritized: first treating sleep problems and then assessing its effect on psychopathology; first treating psychopathology and then assessing its effect on sleep problems; or implementing a combined treatment since the very beginning [30]. Sleep problems in adolescents are known to interfere with depression treatment and may compromise its outcomes [31]. For this reason, it has been suggested that first treating sleep could improve the functional outcomes of treatment of the underlying psychopathology, especially affective symptoms in adolescents [32], although further studies are needed. Among nonpharmacological approaches, cognitive behavioral therapy (CBT) combined with mindfulness-based interventions has proven more effective than traditional CBT for insomnia and mood–anxiety disorders as it acts on shared mechanisms [33] by reducing hypervigilance and hyperarousal which are common to both conditions. On the other hand, guidelines concerning the specific drug treatment of sleep problems in comorbidity with psychiatric disorders are still less clear and more research is paramount to assess the long-term effectiveness of pharmacological interventions. Evidence suggests that melatonin is particularly effective to improve sleep quality in youths with comorbid psychiatric disorders and neurodevelopmental conditions, particularly for those with evening chronotypes and sleep phase delay [34,35,36,37]. However, melatonin should be used with caution among young people, as concentrations of over-the-counter melatonin can vary widely compared to product labels. Furthermore, over-ingestion, even accidentally, can have adverse effects. Like other pharmacological interventions, additional research is warranted.

## 5. Strengths and Limitation

The present study provides valuable insights into the complex relationship between sleep, ED, and psychopathology in youth. It thoroughly examines both subjective sleep quality and emotional regulation across a clinical sample of adolescents diagnosed with several psychiatric disorders and neurodevelopmental conditions by using a comprehensive set of standardized clinical measures. The inclusion of drug-naïve patients and the application of statistical models that take into account age and gender as covariates ensures the reliability of the findings, shedding light on potential relationships and highlighting the clinical relevance of addressing sleep quality in psychiatric treatment.

However, several limitations should be acknowledged. First, the study design relies on correlational analyses, thus limiting the ability to establish causal links between sleep and ED variables. Longitudinal studies could provide further insights into causality and potential bidirectional influences among these variables. Additionally, the use of self-reported measures for sleep quality and ED may introduce biases related to subjective perceptions and reporting accuracy. Instead, objective measures, such as polysomnography, could be used as a complement to self-reports in order to provide a more comprehensive understanding of sleep pathophysiology. Furthermore, the generalizability of the findings may be limited by the specific characteristics of the clinical sample recruited from a single hospital setting. Future research including populations and settings from multiple centers across countries could enhance the validity of the results. Finally, important variables that could significantly affect sleep quality and behavioral problems also include motor/sport activity and the use of electronic devices, which hence should be taken into consideration for the future studies. Similarly, a control group of healthy adolescents will be paramount to confirm whether these relationships are also present in this group.

## 6. Conclusions

In conclusion, our study contributes significantly to the understanding of how sleep and emotion regulation reciprocally interact in adolescence and psychopathology. By identifying the bidirectional relationship between sleep quality and ED, the present article lays foundations for future research in this area and suggests potential future direction for therapeutic interventions. Ultimately, a better comprehension of these interactions could aid in the development of more targeted and effective treatment approaches for adolescents struggling with emotional dysregulation.

## Figures and Tables

**Table 1:** Clinical characteristics of the sample.

	Patients	Percentages
Bipolar Spectrum Disorders	74	82.22%
Major Depressive Disorder	12	13.33%
Anxiety Disorders		
*Multiple*	52	57.78%
*Isolated*	22	24.44%
Eating Disorder	25	27.78%
Substance Use Disorder	3	3.33%
Personality Disorders		
*DSM-5-based Cluster A*	2	2.22%
*DSM-5-based Cluster B*	24	26.67%
*DSM-5-based Cluster C*	8	8.89%
Autism Spectrum Disorder	6	6.67%
Social Communication Disorder	19	21.11%
ADHD		
*Combined presentation*	36	40.00%
*Inattentive presentation*	22	24.44%
Tics/Tourette Syndrome	5	5.56%
Specific Learning Disabilities	15	16.67%
Previous Suicide Attempts	6	6.67%
Suicidal Ideation	15	16.67%
Non-Suicidal Self-Injury	14	15.56%
	**Mean ± SD**	
WISC-IV–FSIQ/GAI	101.00 ± 16.68	
*Verbal Comprehension Index*	103.15 ± 16.52	
*Perceptual Reasoning Index*	106.63 ± 15.77	
*Working Memory Index*	88.81 ± 16.24	
*Processing Speed Index*	85.45 ± 47.00	

**Table 2:** Sleep questionnaires.

PSQI Subscales	Mean ± SD
Sleep Quality	1.09 ± 0.90
Sleep Latency	1.34 ± 1.06
Sleep Duration	0.67 ± 1.042
Sleep Efficiency	0.51 ± 0.97
Sleep Disturbances	1.36 ± 0.69
Sleep Medications	0.74 ± 1.24
Daytime Dysfunction	1.16 ± 0.97
**BRIAN Subscales**	**Mean ± SD**
Sleep	12.01 ± 4.04
Activity	12.01 ± 4.35
Sociality	9.32 ± 3.22
Eating	8.22 ± 3.16
Total	41.56 ± 12.05
**MEQ Chronotypes**	**Percentage**
Morningness	21.13%
Intermediate	57.75%
Eveningness	21.13%

**Table 3:** Emotional Dysregulation questionnaires.

Questionnaire	Subscale	Patients	Percentage
**CBCL—6/18**	Deficient Emotional Self-Regulation	31	39.74%
	Dysregulation Profile	34	43.59%
**YSR**	Deficient Emotional Self-Regulation	36	49.32%
	Dysregulation Profile	15	20.55%
**RIPoST-Y**	Affective Instability	32	45.07%
	Emotional Reactivity	40	56.34%
	Interpersonal Sensitivity	23	32.39%
**ARI**	Total	41	59.42%

**Table 4:** Effects of emotional dysregulation on sleep variables.

*PSQI*	Sum of Squares	df	Mean Square	F	*p*	η²
RIPoSt-Y AI groups	56.661	1	56.661	3.976	0.050 *	0.058
RIPoSt-Y ER groups	7.999	1	7.999	0.561	0.457	0.008
RIPoSt-Y IS groups	0.124	1	0.124	0.009	0.926	0.000
Age Range	68.195	1	68.195	4.785	0.033 *	0.070
Gender	3.780	1	3.780	0.265	0.608	0.004
Residuals	840.868	59	14.252			
***BRIAN***	**Sum of Squares**	**df**	**Mean Square**	**F**	***p***	**η²**
RIPoSt-Y AI groups	369.143	1	369.143	4.251	0.044 *	0.057
RIPoSt-Y ER groups	798.515	1	798.515	9.196	0.004 *	0.123
RIPoSt-Y IS groups	0.919	1	0.919	0.011	0.918	0.000
Age Range	283.035	1	283.035	3.260	0.076	0.044
Gender	4.778	1	4.778	0.055	0.815	0.000
Residuals	5036.316	58	86.833			
***MEQ***	**Sum of Squares**	**df**	**Mean Square**	**F**	***p***	**η²**
RIPoSt-Y AI groups	312.593	1	312.593	3.065	0.085	0.050
RIPoSt-Y ER groups	58.619	1	58.619	0.575	0.452	0.009
RIPoSt-Y IS groups	40.761	1	40.761	0.400	0.530	0.007
Age Range	0.042	1	0.042	0.0004	0.984	0.000
Gender	0.553	1	0.553	0.005	0.942	0.000
Residuals	5813.919	57	101.999			

Abbreviations: AI = affective instability; BRIAN = Biological Rhythms Interview of Assessment in Neuropsychiatry; ER = emotional reactivity; IS = interpersonal sensitivity; MEQ = Morningness Eveningness Questionnaire; PSQI = Pittsburgh Sleep Quality Index; RIPoSt-Y = Reactivity, Intensity, Polarity and Stability questionnaire—Youth version. * *p* < 0.05.

**Table 5:** Effects of sleep quality on clinical variables.

*CBCL—DPI*	Sum of Squares	df	Mean Square	F	*p*	η^2^
PSQI groups	0.337	1	0.337	0.000	0.983	0.000
Age Range	63.769	1	63.769	0.091	0.764	0.001
Gender	3929.490	1	3929.490	5.611	0.021 *	0.085
Residuals	42,016.650	60	700.277			
***CBCL—Internalizing***	**Sum of Squares**	**df**	**Mean Square**	**F**	***p***	**η^2^**
PSQI groups	16.600	1	16.600	0.232	0.632	0.004
Age Range	125.397	1	125.397	1.750	0.191	0.027
Gender	165.754	1	165.754	2.313	0.134	0.036
Residuals	4300.445	60	71.674			
***CBCL—Externalizing***	**Sum of Squares**	**df**	**Mean Square**	**F**	***p***	**η^2^**
PSQI groups	0.646	1	0.646	0.004	0.948	0.000
Age Range	25.592	1	25.592	0.173	0.679	0.003
Gender	348.832	1	348.832	2.355	0.130	0.038
Residuals	8887.250	60	148.121			
***YSR—DPI***	**Sum of Squares**	**df**	**Mean Square**	**F**	***p***	**η^2^**
PSQI groups	1378.261	1	1378.261	2.489	0.120	0.039
Age Range	3256.052	1	3256.052	5.880	0.019 *	0.092
Gender	428.168	1	428.168	0.773	0.383	0.012
Residuals	30,454.419	55	553.717			
***YSR—Internalizing***	**Sum of Squares**	**df**	**Mean Square**	**F**	***p***	**η^2^**
PSQI groups	657.356	1	657.356	6.569	0.013 *	0.085
Age Range	1210.765	1	1210.765	12.100	<0.001 *	0.157
Gender	326.821	1	326.821	3.266	0.076	0.042
Residuals	5503.483	55	100.063			
***YSR—Externalizing***	**Sum of Squares**	**df**	**Mean Square**	**F**	***p***	**η^2^**
PSQI groups	36.655	1	36.655	0.354	0.554	0.006
Age Range	3.654	1	3.654	0.035	0.852	0.000
Gender	22.530	1	22.530	0.218	0.643	0.004
Residuals	5693.400	55	103.516			
***ARI—Total***	**Sum of Squares**	**df**	**Mean Square**	**F**	***p***	**η^2^**
PSQI groups	2.161	1	2.161	0.192	0.663	0.004
Age Range	24.551	1	24.551	2.182	0.146	0.042
Gender	1.766	1	1.766	0.157	0.694	0.003
Residuals	562.669	50	11.253			

Abbreviations: ARI = Affective Reactivity Index; CBCL = Child Behavior Checklist; DPI = Dysregulation Profile Index; PSQI = Pittsburgh Sleep Quality Index; YSR = Youth Self Report. * *p* < 0.05.

**Table 6:** Effects of chronotypes on clinical variables.

*CBCL—DPI*	Sum of Squares	df	Mean Square	F	*p*	η^2^
MEQ group	1557.099	2	778.550	1.155	0.322	0.035
Age Range	98.139	1	98.139	0.146	0.704	0.002
Gender	3924.709	1	3924.709	5.821	0.019 *	0.089
Residuals	38,431.911	57	674.244			
***CBCL—Internalizing***	**Sum of Squares**	**df**	**Mean Square**	**F**	***p***	**η^2^**
MEQ groups	156.692	2	78.346	1.126	0.331	0.035
Age Range	208.511	1	208.511	2.997	0.089	0.047
Gender	121.227	1	121.227	1.742	0.192	0.027
Residuals	3965.841	57	69.576			
***CBCL—Externalizing***	**Sum of Squares**	**df**	**Mean Square**	**F**	***p***	**η^2^**
MEQ groups	110.082	2	55.041	0.363	0.697	0.012
Age Range	18.048	1	18.048	0.119	0.731	0.002
Gender	325.885	1	325.885	2.149	0.148	0.036
Residuals	8642.144	57	151.617			
***YSR—DPI***	**Sum of Squares**	**df**	**Mean Square**	**F**	***p***	**η^2^**
MEQ groups	1271.879	2	635.939	1.128	0.332	0.035
Age Range	5621.108	1	5621.108	9.967	0.003 *	0.154
Gender	364.139	1	364.139	0.646	0.425	0.010
Residuals	29,326.800	52	563.977			
***YSR—Internalizing***	**Sum of Squares**	**df**	**Mean Square**	**F**	***p***	**η^2^**
MEQ groups	351.441	2	175.721	1.606	0.210	0.042
Age Range	2072.273	1	2072.273	18.940	<0.001 *	0.247
Gender	284.199	1	284.199	2.598	0.113	0.034
Residuals	5689.328	52	109.410			
***YSR—Externalizing***	**Sum of Squares**	**df**	**Mean Square**	**F**	***p***	**η^2^**
MEQ groups	259.625	2	129.813	1.254	0.294	0.046
Age Range	1.516	1	1.516	0.015	0.904	0.000
Gender	61.826	1	61.826	0.597	0.443	0.011
Residuals	5381.022	52	103.481			
***ARI—Total***	**Sum of Squares**	**df**	**Mean Square**	**F**	***p***	**η^2^**
MEQ groups	3.987	2	1.993	0.172	0.843	0.007
Age Range	20.608	1	20.608	1.775	0.189	0.035
Gender	1.862	1	1.862	0.160	0.691	0.003
Residuals	557.134	48	11.607			

Abbreviations: ARI = Affective Reactivity Index; CBCL = Child Behavior Checklist; DPI = Dysregulation Profile Index; MEQ = Morningness Eveningness Questionnaire; YSR = Youth Self Report. * *p* < 0.05.

## Data Availability

The raw data supporting the conclusions of this article will be made available by the authors on request.
